# Association between preoperative self-rated health and opioid use 12 months after total hip arthroplasty for osteoarthritis: a cohort study using Danish National Health Survey Data

**DOI:** 10.2340/17453674.2025.44758

**Published:** 2025-09-30

**Authors:** Alma B PEDERSEN, Nina M EDWARDS, Maaike G J GADEMAN, Inger MECHLENBURG, Heidi A R JENSEN, Henrik T SØRENSEN

**Affiliations:** 1 Department of Clinical Epidemiology, Aarhus University Hospital, Aarhus; 2 Department of Clinical Medicine, Aarhus University, Aarhus; 3 Department of Orthopaedic Surgery, Aarhus University Hospital, Aarhus; 4 Department of Orthopaedic Surgery, Regional Hospital Horsens, Horsens, Denmark; 5 Department of Orthopaedics, Department of Clinical Epidemiology, Leiden University Medical Center, the Netherlands; 6 Department of Public Health, Aarhus University, Aarhus; 7 Research Centre for Activity and Prevention, VIA University College, Aarhus; 8 National Institute of Public Health, University of Southern Denmark, Copenhagen; 9 Center for Population Medicine, Aarhus University, Aarhus, Denmark

## Abstract

**Background and purpose:**

We examined the association between preoperative self-rated health (SRH) and opioid use 12 months after total hip arthroplasty (THA) for osteoarthritis.

**Methods:**

We identified 381,323 people who answered a question on SRH in the Danish National Health Surveys 2010, 2013, or 2017. Among these, 4,174 people age > 35 years subsequently underwent THA for osteoarthritis. SRH was categorized as poor (“poor” or “fair” health) or good (“good,” “very good,” or “excellent” health). Opioid use was defined as ≥ 2 prescriptions 1–12 months after THA. We calculated prevalences and prevalence ratios (PR) with 95% confidence intervals (CI) through log-binomial regression, overall and by preoperative opioid use status adjusting for potential confounders. The total morphine milligram equivalent (MME) dose after THA with interquartile range (IQR) was further calculated.

**Results:**

876 (21%) patients rated their health as poor and 3,292 (79%) as good. The prevalence of opioid use among patients with poor SRH was higher than among those with good SRH (PR 2.33, CI 2.05–2.65) (315 [36%] vs 132 [14%]). Similarly, among preoperative non-users, the prevalence was 62 (15%) for patients with poor SRH and 140 (6%) for patients with good SRH (PR 2.20, CI 1.65–2.93), and among preoperative users, the prevalence was 252 (54%) for patients with poor SRH and 299 (31%) for patients with good SRH (PR 1.64, CI 1.44–1.86). The overall median MME dose was higher among patients with poor SRH (2,940, IQR 800–9,610) than among those with good SRH (1,000, IQR 400–3,175) with a median difference of 1,940 (IQR 1,227–2,653).

**Conclusion:**

Compared with good preoperative SRH, poor preoperative SRH was associated with higher opioid use 12 months after THA for osteoarthritis.

Opioids are used in managing short-term postoperative pain among patients undergoing total hip arthroplasty (THA) [1]. Despite advancements in perioperative pain control techniques, including regional anesthesia, fast-track surgery, multimodal analgesia, and implementation of tapering plans post-discharge [2], opioid use during THA recovery remains concerning. As many as 60% of patients are prescribed opioids multiple times in the first year after arthroplasty [3-7]. In Denmark, approximately 5–6% of THA opioid-naive patients redeem opioid prescriptions at least twice within 1 year after surgery [8]. Opioid use has several adverse effects on quality of life and the recovery process, including the development of complications, addiction, and diminished survival rates, thus straining healthcare resources in most countries [9].

Self-rated health (SRH), from the individual’s own perspective, is a widely used indicator of current health status [10,11]. It encompasses physical, mental, and social functioning [12]. SRH reflects coping resources and influences health-related behaviors that consequently affect outcomes. Indeed, SRH is associated with morbidity, including the development of diabetes and cardiovascular diseases [13,14], and mortality in a range of populations [15]. In addition, it is associated with increased prescription of drugs, such as analgesics in older people [16]. Two studies have associated SRH with postoperative pain and analgesic use [17,18]. However, large observational cohort studies examining the effect of SRH on opioid use after THA remain lacking. Preoperative SRH might serve as a simple tool, using easily obtainable data, to facilitate risk stratification and identification of patients at high risk of opioid use after THA.

We examined the association between preoperative SRH and opioid use after THA in patients with hip osteoarthritis.

## Methods

### Study design

We conducted a population-based cross-sectional study using data from nationwide Danish health surveys and medical databases. The Danish National Health Service provides tax-supported healthcare for the entire Danish population, ensuring universal access to all hospitals and primary medical care.

The study is presented according to the Strengthening the Reporting of Observational Studies in Epidemiology guidelines (STROBE).

### Data sources

We compiled data from the following 6 sources:

1. The Danish National Health Survey is conducted among the general population using self-reported questionnaires, either paper-and-pencil or web-based. In 2010, 2013, and 2017, region- and municipality-stratified random samples of individuals aged 16 years or older were invited to participate in the surveys. Eligible participants were identified through the Danish Civil Registration System. Approximately 60% of invited individuals completed a detailed questionnaire containing a minimum of 52 questions on, e.g., morbidity, physical activity, anthropometry, health promotion and prevention, contact with health services, social relations, and socioeconomic factors [19].
2. The Danish Hip Arthroplasty Register, established in 1995, contains clinical information on primary THA. The completeness of registration of primary THA exceeds 90% [20].
3. The Danish National Prescription Registry contains information on all prescriptions dispensed from community pharmacies since 1995, coded according to the Anatomical Therapeutic Chemical (ATC) Classification System [21]. The registry does not include information on hospital prescription dispensing.
4. The Danish National Patient Registry contains data on all somatic inpatient admissions to Danish hospitals since 1977 and all outpatient clinic visits and emergency department visits since 1995. Each record is linked to the patient’s civil registration number and includes information on the treatments and surgical procedures performed, as well as 1 primary and as many as 19 secondary discharge diagnoses. The discharge diagnoses are coded according to the International Classification of Diseases, Eighth Revision (ICD-8; from 1977 to 1993) and Tenth Revision (ICD-10; starting in 1993) [22].
5. The Population Education Register contains individual-level information on the education obtained by each Danish citizen [23].
6. The Danish Civil Registration System has assigned a unique civil registration number to all Danish citizens at birth or immigration since 1968. This number enables unambiguous individual-level record linkage across multiple databases and surveys and provides information on vital status and migration, thereby ensuring complete follow-up [24].

### Study population

Inclusion criteria: (i) Invited to participate in the Danish National Health Survey in 2010, 2013 and 2017; (ii) Responded to a questionnaire including a question on SRH; (iii) Age above 35 years; (iv) Undergoing THA due to osteoarthritis after answering SRH question; (v) Alive at day 30 after THA.

Exclusion criteria: (i) Not responded to invitation from the Danish National Health Surveys; (ii) Age below 35 years; (iii) No THA due to osteoarthritis after answering SRH question; (iv) Died within 30 days of THA.

The final study population consisted of 4,168 patients.

### Self-rated health

SRH was measured with a single-item question: “How do you rate your present state of health in general?” Individuals rated their health status on a 5-point Likert scale. According to the answers, patients undergoing THA were divided into 1 of 2 exposure groups: poor SRH (“poor” or “fair” health) or good SRH (“good,” “very good,” or “excellent” health). In a few cases in which a patient answered the SRH question multiple times before THA, the answer closest to the THA was used.

### Opioids and outcome

Information on dispensing of 13 common types of opioids was collected from the Danish National Prescription Registry, according to ATC codes. The primary outcome was opioid use, defined as dispensing of ≥ 2 opioids within 1–12 months after THA [25-27]. Dispensing of opioids during the 0–30-day postoperative period was excluded because this practice was considered part of standard pain management after THA surgery.

We also defined opioid use as the total number of dispensing occasions 1–12 months after THA.

Because doses vary among prescriptions of different opioid types, all doses were converted to milligram morphine equivalent (MME) doses with a conversion factor corresponding to each specific opioid type [28]. Doses and conversion factors were available for > 95% of all included prescriptions and for > 99% of the 6 most common opioids.

Patients redeeming at least 1 opioid prescription within 0–6 months before the THA procedure were defined as preoperative opioid users.

### Covariates

We collected information on the following covariates at the time of SRH status allocation:

1. Patient age and sex were collected from the Civil Registration System.
2. Somatic comorbidities 2-year history was obtained from the Danish National Patient Registry. According to ICD codes, we calculated a Charlson Comorbidity Index (CCI) score for every patient. The comorbidity level was classified into 3 categories: low (CCI score 0), moderate (CCI score 1–2), or high (CCI score ≥ 3).
3. The highest level of education attained was determined from the Population Education Register and classified as low (primary or lower secondary education), moderate (vocational education and training, qualifying educational programs, upper secondary education, or short-cycle tertiary education), or high (bachelor’s programs, master’s programs, and PhD programs or higher).
4. Pain-related conditions history was obtained from the Danish National Patient Registry according to ICD codes for chronic musculoskeletal pain, neuropathic pain, cancer-related pain, headache and orofacial pain, and postsurgical pain [29].
5. Mental disorders 2-year history was obtained from the Danish National Patient Registry according to ICD codes [30].
6. Medication use (defined as at least 1 prescription 1 year before SRH) was obtained from the Danish National Prescription Registry, according to ATC codes for non-steroid anti-inflammatory drugs, steroids, statins, and chronic obstructive pulmonary disease drugs.

### Statistics

Patient characteristics were described according to their SRH status.

For the overall study population, we calculated the prevalence of opioid use according to SRH. Differences in prevalence between patients with poor vs good SRH (reference) were determined for patients who were alive at the start of day 30 after THA. Thus, follow-up began 30 days after THA surgery. Using log-binomial regression, we calculated crude prevalence differences, crude prevalence ratios, and prevalence ratios adjusted for age, sex, CCI, mental disorders, medication use, and education, for patients with poor SRH vs good SRH.

The total MME per person 1–12 months after THA and the median among all patients who redeemed an opioid in the same period were further calculated with interquartile range (IQR). The median difference with 95% confidence intervals (CI) was calculated with bootstrapping.

In addition, to examine the dose–response association between SRH and opioid use after THA, we estimated adjusted prevalence ratios for patients with poor, fair, good, or very good SRH vs patients with excellent SRH in the overall study population.

Prevalences, prevalence differences and ratios, and MME were further calculated by preoperative opioid use status. All estimates are presented with CIs.

Finally, to test for immortal time bias and selection bias we calculated mortality by SRH 1 year after answering SRH and further restricted overall analyses to individuals who underwent THA within 1 year after answering SRH. The mortality by SRH for 1 year follow-up after THA was also reported to evaluate the potential selection bias.

The analyses were performed in Stata 17.0 (StataCorp LLC, College Station, TX, USA).

### Handling of missing data

Approximately 60% of invited persons completed a detailed questionnaire in the Danish National Health Survey, with lower response rate among persons with lower educational status [22]. Although we have previously shown that responders are highly representative of the overall THA population [31], it is likely that some THA patients with low education were not captured. As low education is associated with both poor SRH and opioid use after THA, our risk estimates could be affected. To reduce differential responders bias, survey weighting methods could be applied. However, we were unable to use this approach because data on non-responders was not available for us from Denmark Statistics for this study. Previous studies have shown a small difference in absolute risk estimates between a weighted and an unweighted survey sample [32].

Less than 2% of our final study population had missing data on education, and because the missingness was not random, we did not perform multiple imputation.

### Ethics, funding, and disclosures

The study was reported to the Danish Data Protection Agency through registration at Aarhus University (record number: AU-2016-051-000001, sequential number 880). Ethical approval is not required in Denmark for studies on routinely collected registry data. Individuals participating in the Danish National Health Surveys have participated voluntarily and have given written permission that their data can be used for research purposes. According to the Danish legislation, datasets generated and/or analyzed in the current study are not publicly available. ABP and HTS were supported by, and employed at, the Department of Clinical Epidemiology at Aarhus University, and Aarhus University Hospital. The Department of Clinical Epidemiology, Aarhus University, and Aarhus University Hospital receive funding from various companies in the form of research grants to (and administered by) Aarhus University. None of these grants are related to the present study. The authors declare no conflicts of interest. Complete disclosure of interest forms according to ICMJE are available on the article page, doi: 10.2340/17453674.2025.44758

## Results

### Population

**Figure 1:**
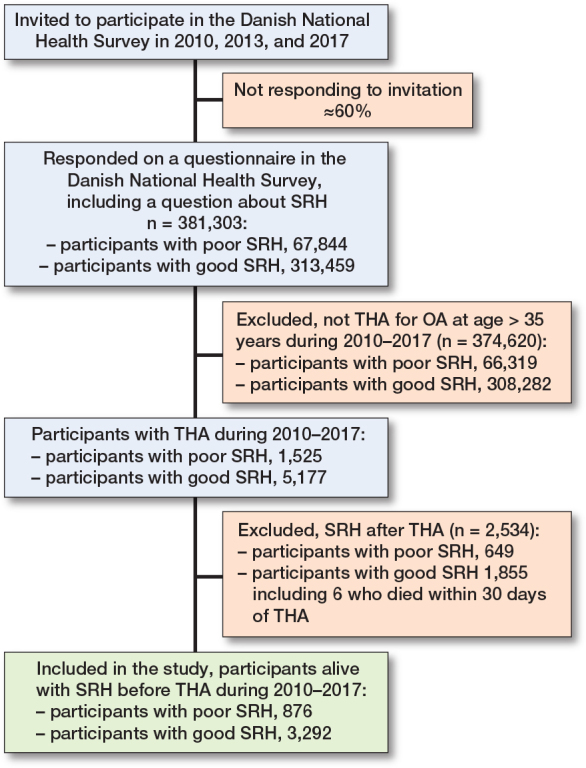
Flow diagram. THA: total hip arthroplasty.

We identified a cohort of 381,323 respondents who completed the question on SRH in the Danish National Health Surveys in 2010, 2013, or 2017. Within this cohort, we identified 4,174 people age > 35 years, who, after answering the question on SRH, underwent THA between 2010 and 2018, as recorded in the Danish Hip Arthroplasty Registry. After exclusion of 6 patients who died during 0–30 days of follow-up (corresponding to the start of outcome measurement at day 30), the final study population consisted of 4,168 patients (Figure 1).

876 (21%) THA patients had poor SRH, while 3,292 (79%) had good SRH before THA. The median number of days from completion of the question on SRH and THA was 1,084 days overall (IQR 426–1,780), 780 days (IQR 286–1,548) for patients with poor SRH, and 1,160 days (IQR 515–1,826) for patients with good SRH.

Patients with poor and good SRH had similar median ages. Compared with patients with good SRH, patients with poor SRH were more likely to be female (60% vs 54%), to have moderate or high CCI scores (49% vs 27%), to have pain-associated conditions (14% vs 7%), to take various medications (87% vs 79%), and to have low education (40% vs 31%) (Table 1).

**Table 1:** Characteristics of the study population, overall and according to preoperative opioid use

Characteristic	Overall		Preoperative		Preoperative	
			opioid non-users		opioid users^a^	
	Poor SRH	Good SRH	Poor SRH	Good SRH	Poor SRH	Good SRH
	n = 876	n = 3,292	n = 410	n = 2,328	n = 466	n = 964
Female sex	529 (60)	1,789 (54)	233 (57)	1,222 (52)	296 (64)	567 (59)
Age, years						
< 60	139 (16)	470 (14)	75 (18)	359 (15)	64 (14)	111 (12)
60–70	274 (31)	1,081 (33)	141 (34)	817 (35)	133 (29)	264 (27)
> 70	463 (53)	1,741 (53)	194 (47)	1,152 (49)	269 (58)	589 (61)
Charlson Comorbidity Index						
Low	451 (51)	2,392 (73)	237 (58)	1,768 (76)	214 (46)	624 (65)
Moderate	303 (35)	739 (22)	129 (31)	470 (20)	174 (37)	269 (28)
High	122 (14)	161 (4.9)	44 (11)	90 (3.9)	78 (17)	71 (7.4)
Education						
Low	348 (40)	1,007 (31)	149 (36)	680 (29)	199 (43)	327 (34)
Moderate	374 (43)	1,450 (44)	180 (44)	1,028 (44)	194 (42)	422 (44)
High	142 (16)	794 (24)	75 (18)	596 (26)	67 (14)	198 (21)
Missing	12 (1.4)	41 (1.3)	6 (1.5)	24 (1.0)	6 (1.3)	17 (1.8)
Pain-related conditions	121 (14)	219 (6.7)	36 (8.8)	136 (5-8)	85 (18)	83 (8.6)
Mental disorders	15 (1.7)	20 (0.6)	7 (1.7)	14 (0.6)	8 (1.7)	6 (0.6)
Medication use	764 (87)	2,614 (79)	345 (84)	1,740 (75)	419 (90)	874 (91)

SRH = self-rated health.

a Preoperative opioid users: patients who redeemed at least 1 opioid prescription 0–6 months before THA.

### Opioid use

**Figure 2:**
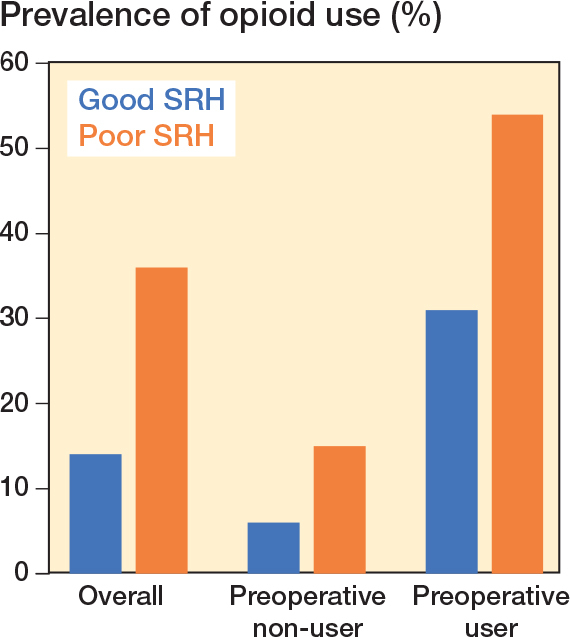
Prevalence of opioid use from 1 month to 1 year after total hip arthroplasty, according to self-rated health (SRH) and preoperative opioid use status. Preoperative users: patients who redeemed at least 1 opioid prescription 0–6 months before THA.

The overall prevalence of opioid use was 36% among patients with poor SRH and 14% among patients with good SRH (Figure 2), corresponding to a prevalence difference of 22.0 (CI 18.6–25.4) percentage points (see Table 1). Overall, the adjusted prevalence ratio for postoperative opioid use was 2.33 (CI 2.05–2.65) for patients with poor SRH vs patients with good SRH. The median number of opioid prescriptions dispensed 1–12 months after THA was 4 (IQR 2–9) among patients with poor SRH and 2 (IQR 1–5) among patients with good SRH. The median MME dose 1–12 months after THA was 2,940 mg (IQR 800–9,610) among patients with poor SRH and 1,000 mg (IQR 400–3,175) among patients with good SRH corresponding to a median difference of 1,940 (1,227–2,653). A dose–response association was observed between SRH level and opioid use: the prevalence ratios were 1.24 (0.73–2.12) among patients with very good SRH, 2.03 (1.21–3.38) among patients with good SRH, 4.24 (2.54–7.08) among patients with fair SRH, and 6.35 (3.71–10.86) among patients with poor SRH, as compared with patients with excellent SRH.

Among the 4,168 THA patients alive on day 30 from THA, 1,430 (34%) were preoperative opioid users, and 2,738 (66%) were preoperative opioid non-users. A similar distribution of patient characteristics by SRH was observed among preoperative opioid users and non-users, while preoperative opioid users in general had worse health than non-users (see Table 1).

**Figure 3:**
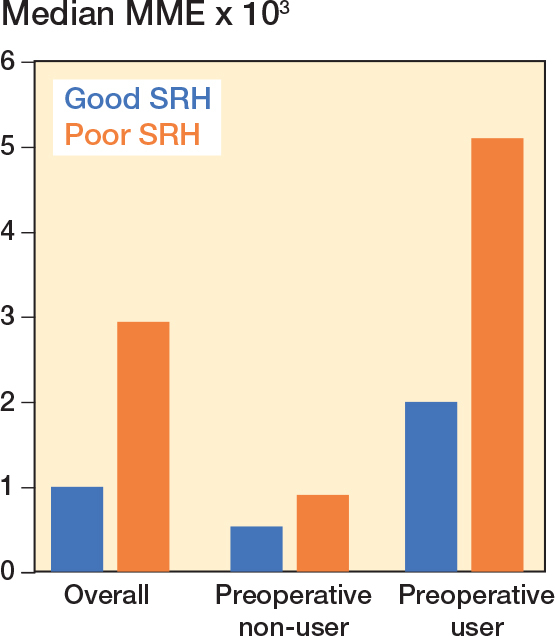
Median milligram morphine equivalent dose (MME) from 1 month to 1 year after total hip arthroplasty, according to self-rated health (SRH) and preoperative opioid use status. Preoperative users: see Figure 2.

For preoperative non-users, the prevalence of opioid use was 15% among patients with poor SRH and 6% among patients with good SRH. This corresponds to a prevalence difference of 8.2 (CI 4.6–11.8) percentage points and the adjusted prevalence ratio of 2.20 (CI 1.65–2.93) for those with poor SRH vs good SRH (Table 2). For preoperative users, the prevalence of continuous opioid use was 54% among patients with poor SRH and 31% among patients with good SRH. This corresponds to a prevalence difference of 23.1 (CI 17.7–28.5) percentage points and the adjusted prevalence ratio was 1.64 (CI 1.44–1.86) for those with poor SRH vs good SRH (see Table 2). The same difference in MME by SRH was observed among preoperative opioid non-users and users, and the median MME was notably higher among preoperative users (Figure 3).

**Table 2:** Prevalence and prevalence ratios of opioid use from 1 month to 1 year after total hip arthroplasty

	Prevalence, n (%)		Crude PD in percentage points (CI)	Crude PR (CI)	Adjusted PR^a^ (CI)
	Good SRH	Poor SRH			
Overall	132 (14)	315 (36)	22.0 (18.6–25.4)	2.61 (2.31–2.95)	2.33 (2.05–2.65)
Preoperative opioid non-users	140 (6)	62 (15)	8.2 (4.6–11.8)	2.27 (1.72–3.01)	2.20 (1.65–2.93)
Preoperative opioid users	299 (31)	252 (54)	23.1 (17.7–28.5)	1.74 (1.53–1.97)	1.64 (1.44–1.86)

SRH = self-rated health. CI = 95% confidence interval. PD = prevalence difference. PR = prevalence ratio.

Preoperative opioid users: patients who redeemed at least 1 opioid prescription 0–6 months before THA.

a Adjusted for age, sex, Charlson Comorbidity Index, mental disorders, medication use, and education.

Restricting to individuals who underwent THA within 1 year of reporting SRH (n = 900), the prevalence difference for postoperative opioid use was 16.9 (CI 11.0–22.7) percentage points and the adjusted prevalence ratio was 2.26 (CI 1.67–3.06) for patients with poor SRH vs patients with good SRH. Among preoperative opioid non-users the prevalence difference was 2.0 (CI –2.8 to 6.9) percentage points and the adjusted prevalence ratio was 1.15 (CI 0.57–2.33) for those with poor SRH vs good SRH, while among preoperative opioid users the prevalence difference was 25.5 (CI 14.8–36.3) percentage points and the adjusted prevalence ratio was 1.94 (CI 1.43–2.62) for those with poor SRH vs good SRH.

### Mortality

The mortality within 1 year of answering SRH in 2010 was 3.8% among people with poor and 0.5% among people with good SRH. A similar difference in mortality was observed among individuals answering SRH in 2013 and 2017.

The mortality rate 1–12 months after THA was 1.03% (CI 0.47–1.94) and 0.76% (CI 0.49–1.12) among patients with poor and good SRH, respectively.

## Discussion

We examined the association between preoperative SRH and opioid use after THA in patients with hip osteoarthritis. We found that patients with poor preoperative SRH are more likely to experience pain and to use opioids for 1–12 months after THA compared with those with good SRH. Furthermore, patients with poor SRH are more likely to redeem higher doses of opioids than patients with good SRH.

### Comparison with other studies and clinical implications

A study including 970 opioid-naïve patients [17] showed that approximately 14% of orthopedic patients were taking opioids after 90 days. Fair/poor SRH vs good/very good/excellent SRH was associated with a 1.4-fold adjusted increased risk of opioid use. Most patients (80%) reported that their main reason for opioid use after surgery was to mitigate pain, whereas 6% cited improved sleep, and 11% cited other unspecified reasons. This finding is noteworthy considering the limitations of our registry data, for which the lack of indication for opioid prescription is often noted as a notable limitation. However, if 80% of indications for opioid use were for pain relief, this limitation might not be substantial.

Studies in various populations have demonstrated that SRH is an independent predictor of mortality [15], regardless of age, sex, and the presence of chronic illness [33] and multimorbidity [13,14,34]. The association between SRH and mortality is further influenced by lifestyle factors and socioeconomic position; although this association is reduced after adjusting for these factors, it does not disappear entirely [35]. Our results showing an association between poor SRH and opioid use—even after adjusting for relevant confounders—are consistent with the findings from these mortality studies. The finding that 34% of THA patients were prescribed opioids within 6 months before surgery is consistent with existing literature. This may be explained by the fact that patients awaiting surgery often experience severe pain that can only be managed with opioids. These patients are likely aware that surgery is imminent and that opioid use will probably cease postoperatively.

Several possible explanations exist for the association between SRH and opioid use after THA, including comorbidities, social networks, and postoperative complications. Although we adjusted for comorbidities, individuals consider a wide spectrum of factors when rating their health [12], some of which might not be immediately evident to healthcare professionals or fully captured by standard health indicators. The SRH incorporates many aspects of health-related behavior; mental health; and social, economic, political, and environmental factors influencing health [12]. In theories of health [36], bodily perceptions can change in response to disease and health experiences. The theory of allostasis [37] suggests that the human body responds to perceived anticipated stressors by activating the autonomic nervous system, and various hormonal cascades and immune reactions leading to allostatic load [38] and poorer health outcomes. Through SRH, patients might be able to detect allostatic load and preclinical conditions requiring opioid treatment long before they experience actual symptoms of specific conditions. Previous research links social disconnectedness and perceived isolation to lower levels of SRH among older people [39]. Poor SRH is also likely to be associated with an increased risk of post-THA complications, as has been suggested in patients undergoing cardiac surgery [40] necessitating opioid use.

Several studies have reported an association between chronic pain and poor SRH [18,41]. Because our study included patients with hip osteoarthritis and worsening joint pain before THA, the patients might potentially have had initial chronic pain due to osteoarthritis, which led to their poor SRH and subsequently contributed to increased opioid use after THA. Thus, the patients might have experienced a vicious circle, in which pain promoted poor health, and vice versa.

### Limitations

A cross-sectional study design is less suitable for causal questions. There could be some immortal time bias from the time of measuring SRH to THA because people with poor SRH have a higher chance of dying and never being offered THA than those with good SRH, as we have seen in our data. Data on SRH was available only for a small proportion of patients undergoing THA registered in the Danish Hip Arthroplasty Register. However, available patients were largely representative of the entire THA population, except for educational level [31]. Misclassification of SRH was possible, because as many as 1.5 years elapsed between the SRH answer and THA. However, previous research has indicated that SRH measures have good reliability in the Scandinavian context, even when several years elapse between the first and second measurements [42]. Our data shows opioid redemption, not opioid consumption. Therefore, misclassification of opioid use is possible. Residual confounding due to misclassification of comorbidities is possible as we included only comorbidities requiring hospital contact, while those mild cases treated by general practitioners are not included. We adjusted for education, but as less than 2% of patients had missing data on this variable, residual confounding due to education is possible.

### Conclusion

Patients with poor preoperative SRH exhibited higher opioid use and redeemed higher MME doses in the year after THA compared with those with good SRH.

*In perspective,* SRH could be useful for researchers as a confounder variable. Further, SRH could be useful for clinicians as a simple screening tool to identify high-risk patients. Screening of SRH could be incorporated into clinical care to achieve early and rapid identification of patients who might benefit from resource- and time-intensive pain evaluations and treatments. The goal and consequence of screening would differ between opioid-naive patients and opioid users before THA. Future prospective studies on the ability of SRH as a screening tool are warranted. Opioid use as long as 1 year after THA among patients who were not taking opioids before THA is likely to be considered THA failure and a patient safety problem due to the huge patient, family, healthcare, and societal consequences of opioid addiction.

## Notes

ABP and NME contributed to data acquisition. All authors contributed substantially to conception and study design, analyses plan, interpretation of data, drafting and revising the paper, finalizing the paper, final approval of the paper, and are accountable for all aspects of the work.

Handling co-editors: Keijo Mäkelä and Robin Christensen

*Acta* thanks Lars Nordsletten for help with peer review of this manuscript.
